# Dietary Deficiency of Essential Amino Acids Rapidly Induces Cessation of the Rat Estrous Cycle

**DOI:** 10.1371/journal.pone.0028136

**Published:** 2011-11-23

**Authors:** Kazumi Narita, Kenji Nagao, Makoto Bannai, Toru Ichimaru, Sayako Nakano, Takuya Murata, Takashi Higuchi, Michio Takahashi

**Affiliations:** 1 Department of Integrative Physiology, Faculty of Medical Sciences, University of Fukui, Yoshida-gun, Fukui, Japan; 2 Frontier Research Labs, Institute for Innovation, Ajinomoto Co., Inc., Kawasaki-shi, Kanagawa, Japan; Institut de Génomique Fonctionnelle de Lyon, France

## Abstract

Reproductive functions are regulated by the sophisticated coordination between the neuronal and endocrine systems and are sustained by a proper nutritional environment. Female reproductive function is vulnerable to effects from dietary restrictions, suggesting a transient adaptation that prioritizes individual survival over reproduction until a possible future opportunity for satiation. This adaptation could also partially explain the existence of amenorrhea in women with anorexia nervosa. Because amino acid nutritional conditions other than caloric restriction uniquely alters amino acid metabolism and affect the hormonal levels of organisms, we hypothesized that the supply of essential amino acids in the diet plays a pivotal role in the maintenance of the female reproductive system. To test this hypothesis, we examined ovulatory cyclicity in female rats under diets that were deficient in threonine, lysine, tryptophan, methionine or valine. Ovulatory cyclicity was monitored by daily cytological evaluations of vaginal smears. After continuous feeding of the deficient diet, a persistent diestrus or anovulatory state was induced most quickly by the valine-deficient diet and most slowly by the lysine-deficient diet. A decline in the systemic insulin-like growth factor 1 level was associated with a dietary amino acid deficiency. Furthermore, a paired group of rats that were fed an isocaloric diet with balanced amino acids maintained normal estrous cyclicity. These disturbances of the estrous cycle by amino acid deficiency were quickly reversed by the consumption of a normal diet. The continuous anovulatory state in this study is not attributable to a decrease in caloric intake but to an imbalance in the dietary amino acid composition. With a shortage of well-balanced amino acid sources, reproduction becomes risky for both the mother and the fetus. It could be viewed as an adaptation to the diet, diverting resources away from reproduction and reallocating them to survival until well-balanced amino acid sources are found.

## Introduction

Understanding gender-specific nutritional requirements is an important goal of modern health care research, particularly for women whose nutritional requirements change during different reproductive stages, including pregnancy, lactation, menstruation and menopause. Although many of the effects of excess and inadequate dietary energy intake are similar for females and males [1], [2], distinctive sex-dependent responses to dietary energy intake have been reported. For instance, in rodents, caloric restriction inhibits the reproductive cycle in females but does not adversely affect male fertility [3], [4], [5]. Similarly, anorexia nervosa of sufficient severity induces amenorrhea in women [6], [7], whereas no observable reproductive dysfunction is found in men with this eating disorder behavior. Serotonergic vulnerability caused by nutritional insufficiency varies by gender and female reproductive stage [8]. Pre-menopausal women were found to be more resistant than men to obesity-related atherosclerotic heart disease [9], and the influence of energy intake on disease susceptibility may also be different between females and males.

In humans and other species, caloric restriction suppresses the hypothalamic-pituitary-gonadal (HPG) axis by reducing luteinizing hormone (LH) secretion and disrupting ovulatory cyclicity through central inhibition [10], [11], [12]. There have been reports of delayed puberty [13] and reduced fertility [13], [14], [15] in animals that are subjected to caloric restriction. The decrease in available calories is considered the main factor suppressing LH release and ovulatory cyclicity because pharmacological inhibitors of metabolic fuel oxidation, including 2-deoxy-D-glucose, which is a competitive inhibitor of glucose utilization, also disturb the estrous cycle [16], [17], [18]. However, moderate caloric restriction can extend the reproductive lifespan [4], [13], presumably as a result of delaying the aging process.

Recent evidence suggests that many of the central and peripheral endocrine signals that govern energy homeostasis, such as leptin [19], ghrelin [20], [21], polypeptide YY3–36 [22], neuropeptide Y [23], neuromedin U [24], neuromedin S [25] and orexin [26], are involved in the control of reproductive function by acting at different levels of the HPG axis. In addition, Della Torre et al. [27] demonstrated that dietary amino acids regulate the transcriptional activity of hepatic estrogen receptor alpha through a mammalian target of rapamycin (mTOR)-dependent mechanism. In response to hepatic estrogen receptor alpha, insulin-like growth factor 1 (IGF-1) is synthesized in the liver as a molecule that signals nutritional status to the reproductive apparatus in mice. Because these hormone levels [28], [29], [30], [31], [32], including IGF-1 levels [33], [34], are significantly affected by both the protein and amino acid contents of the diet, it is an intriguing question whether changes in the amino acid composition of the diet would influence female reproductive function. Moreover, Grandison et al. [35] recently reported that the dietary amino acid balance strongly affects the fecundity of *Drosophila*; the inhibition of fecundity that is induced by a diet devoid of amino acids was resolved by adding only methionine to the diet, not by the addition of any other single amino acid. This finding indicates that, in addition to the diet's caloric content, the amino acid levels in a diet, either in their absolute amounts or in their balance, can influence reproductive function.

In this study, we investigated the effects of the deficiency of a single essential amino acid on reproductive function in female rats. We examined the changes in estrous cycles under diets that are deficient in threonine (Thr), lysine (Lys), tryptophan (Trp), methionine (Met) or valine (Val). As an essential amino acid-deficient diet has been shown to cause an anorexic effect [29], [30], [36], [37], the specific effects of each amino acid deficiency were evaluated via the appropriate pair feeding experiments. The results of this study are expected to extend our understanding of gender-specific nutritional responses to dietary amino acid imbalances.

## Results

### Body weight and food intake

**Figure 1:**
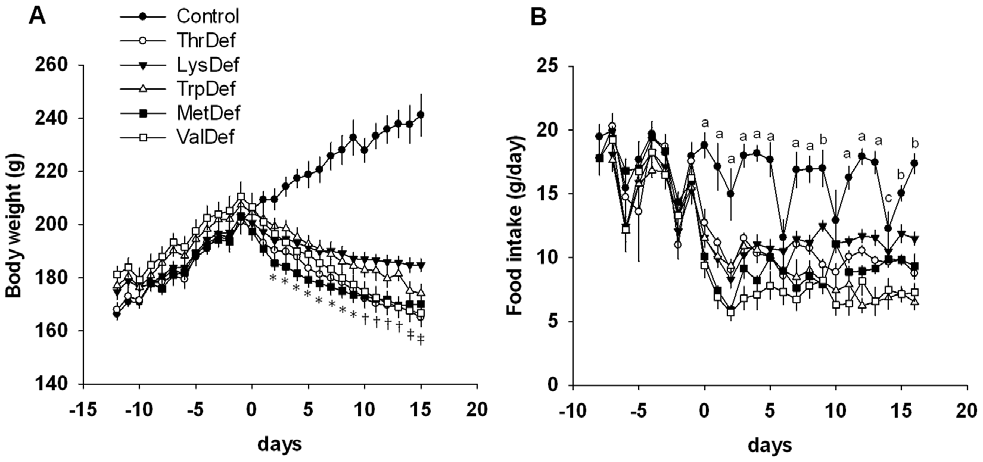
Essential amino acid-deficient diets suppressed food intake and body weight gain. Daily body weight (A) and spontaneous food intake (B) of rats that were fed each essential amino acid-deficient diet are shown. Day 0 corresponds to the first day of the experimental diet. The data are presented as the mean ± SEM. The significant differences (*P*<0.05) are shown using the following symbols or letters. *Control vs. LysDef, TrpDef, ValDef, ThrDef and MetDef. †Control vs. LysDef and TrpDef vs. ValDef, ThrDef and MetDef. ‡Control vs. LysDef vs. TrpDef, MetDef, ValDef and ThrDef. “a” Control vs. LysDef, ThrDef, TrpDef, MetDef and ValDef. “b” Control vs. LysDef vs. ThrDef, TrpDef, MetDef and ValDef. “c” Control, LysDef, MetDef and ThrDef vs. TrpDef and ValDef. N = 4–6.

Body weights and daily food intake are shown in Figures 1A and B, and the food consumption was normalized to body weight in Figure S1. After consuming the experimental diet for 2 days, the essential amino acid-deficient group's average body weight was significantly lower than that of control rats and continued to decline over time. The control rats showed a clear 4-day food intake cycle that was synchronized with the estrous cycle, i.e., the lowest level of food intake occurred on the day of estrous. A significant repression of food intake was observed after the onset of all of the essential amino acid-deficient diets. Cyclic changes in food intake were not detected.

**Figure 2:**
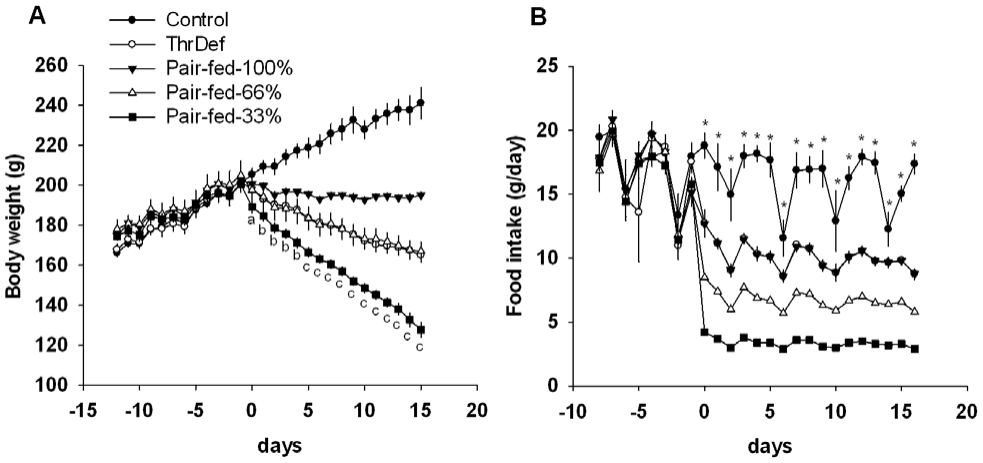
Essential amino acid-deficient diets caused body weight loss under calorie-restricted conditions. (A) and (B) show the daily body weight and food intake, respectively, of rats that were fed the limited amount of control diet in the pair feeding experiment. The Pair-fed-100% rats were offered isocaloric control diets with the same calories as those of the ThrDef group, Pair-fed-66% rats were offered two-thirds that amount, and Pair-fed-33% were offered one-third that amount. The body weight curve of the ThrDef rats was similar to that of the Pair-fed-66% group. Day 0 corresponds to the first day of the experimental diets. The data are presented as the mean ± SEM. The significant differences (*P*<0.05) are indicated by the following letters or symbols: “a” Control vs. Pair-fed-33%. “b” Control vs. Pair-fed-100%, ThrDef and Pair-fed-66% vs. Pair-fed-33%. “c” Control vs. Pair-fed-100% vs. ThrDef and Pair-fed-66% vs. Pair-fed-33%. *Control vs. ThrDef and Pair-fed-100% vs. Pair-fed-66% vs. Pair-fed-33%. N = 4–6.

Figures 2A and B show the average body weight and daily food intake in the pair feeding experiment. Pair-fed-100% rats were offered a control diet that was equal in calories to the amount ingested by the ThrDef group, Pair-fed-66% rats were offered two-thirds as many calories, and Pair-fed-33% rats were offered one-third as many calories. Thus, the food intake levels were significantly different among the three groups. Although the caloric intake of the ThrDef rats was obviously similar to that of the Pair-fed-100% rats, their average body weights were significantly lower than those of their pair feeding counterparts after 6 days on the deficient diet. The change in body weight of the ThrDef rats was most similar to that of Pair-fed-66% rats.

### Biochemical parameters of the plasma and liver

Tables 1 and 2 reveal the liver triglyceride levels and plasma levels of glucose, total cholesterol, triglyceride and NEFA. Intriguingly, the hepatic triglyceride levels tended to become elevated under the LysDef diet, whereas the other essential amino acid-deficient diets decreased the hepatic triglyceride levels. The plasma levels of NEFA were sustained at constant levels regardless of the dietary conditions. The ThrDef and Pair-Fed-100% groups showed altered plasma levels of glucose and triglycerides, but these alterations occurred in opposite directions: the rats on the ThrDef diet had decreased levels, whereas their pair-fed counterparts had increased levels. The levels of plasma glucose and triglycerides were significantly different between the ThrDef and Pair-fed-100% groups.

**Table 1:** Tissue weights and biochemical parameters in Wistar-Imamichi rats fed each essential amino acid deficient diet.

	Body	Ovary	Total fat	Liver	Liver	Plasma	Plasma	Plasma	Plasma
	weight	weight	weight	weight	triglyceride	glucose	total cholesterol	triglyceride	NEFA
	(g)	(%Body weight)	(%Body weight)	(%Body weight)	(mg/g)	(mg/dL)	(mg/dL)	(mg/dL)	(mEq/L)
Ad lib									
Control	240.8±4.0	0.033±0.015	4.6±0.5	3.8±0.2	8.1±1.2	191.8±8.5	84.8±13.6	91.0±14.8	0.23±0.01
LysDef	184.8±2.4*	0.030±0.004	2.5±0.2*	3.1±0.1*	13.1±1.9	178.2±12.8	82.2±3.5	58.4±6.6*	0.23±0.03
TrpDef	174.2±3.4*	0.036±0.003	1.2±0.2*	3.4±0.2	3.7±0.8	167.5±7.4	73.0±8.4	34.3±6.1*	0.29±0.05
MetDef	170.1±3.1*	0.036±0.003	1.0±0.1*	3.6±0.1	3.8±0.0	135.8±5.7*	55.5±2.5*	27.3±4.5*	0.22±0.03
ValDef	166.6±4.2*	0.034±0.001	1.2±0.2*	3.5±0.1	5.3±1.8	154.3±2.1*	86.5±6.6	48.7±4.0*	0.20±0.02
ThrDef	165.1±3.5*	0.035±0.004	1.3±0.1*	3.4±0.2	4.9±1.0	151.8±8.3*	67.5±3.2	28.5±7.2*	0.21±0.01

The data are presented as mean±SEM.

*P<0.05 compared with control group.

**Table 2:** Tissue weights and biochemical parameters in pair-fed Wistar-Imamichi rats.

	Body	Ovary	Total fat	Liver	Liver	Plasma	Plasma	Plasma	Plasma
	weight	weight	weight	weight	triglyceride	glucose	total cholesterol	triglyceride	NEFA
	(g)	(%Body weight)	(%Body weight)	(%Body weight)	(mg/g)	(mg/dL)	(mg/dL)	(mg/dL)	(mEq/L)
Ad lib									
ThrDef	165.1±3.5	0.035±0.004	1.3±0.1	3.4±0.2	4.9±1.0	151.8±8.3	67.5±3.2	28.5±7.2	0.21±0.01
Pair-fed									
100%	193.5±1.9#	0.048±0.002	1.6±0.1	3.6±0.1	3.7±0.5	203.7±6.8#	72.5±6.1	100.2±5.2#	0.20±0.01
66%	165.4±4.5	0.046±0.002	0.5±0.1#	3.2±0.1	0.8±0.2#	171.3±10.8	62.6±1.6	109.2±16.7#	0.19±0.01
33%	122.4±3.7#	0.055±0.006	0.1±0.0#	2.4±0.2#	0.4±0.3#	155.0±18.0	N. D.	30.8±7.9	0.21±0.03

The data are presented as mean±SEM. #P<0.05 compared with ThrDef group. N.D., not detected.

### Hormone levels in the plasma

Table 3 reveals the plasma levels of IGF-1 (F = 27.4, *P*<0.01), leptin (F = 50.1, *P*<0.01), insulin (F = 55.0, *P*<0.01) and desacyl ghrelin (F = 4.4, *P* = 0.03). The plasma levels of IGF-1, leptin and insulin were significantly decreased by the consumption of each essential amino acid-deficient diet, and the plasma desacyl ghrelin level was elevated by the MetDef diet.

**Table 3:** The plasma hormone levels in Wistar-Imamichi rats fed each experimental diet.

	Control	MetDef	ValDef
IGF-1 (ng/mL)	517.22±17.64	189.99±25.38*	206.95±52.57*
Leptin (ng/mL)	12.71±1.64	1.11±0.14*	0.96±0.12*
Insulin (ng/mL)	2.50±0.26	0.42±0.12*	0.28±0.04*
des-acyl ghrelin (fmol/mL)	312.81±38.57	521.91±70.42*	451.62±36.27

The data are present as mean±SEM.

*Significantly different (P<0.05) from control group.

### Estrous cycle

**Figure 3:**
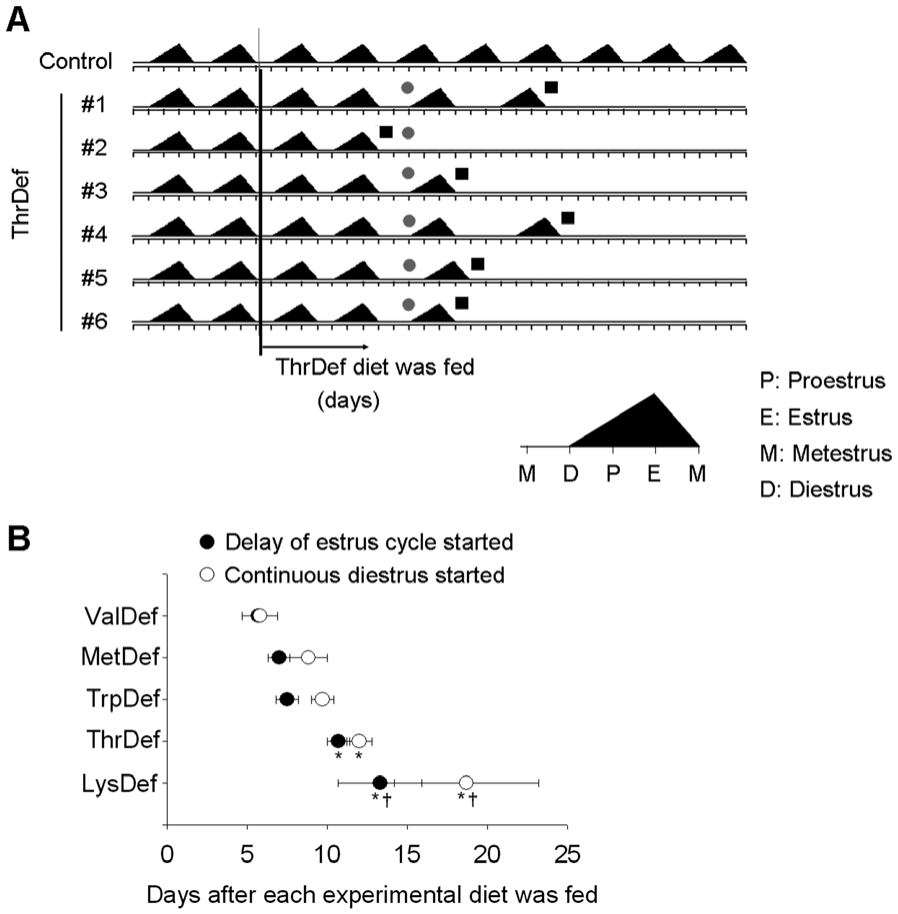
The essential amino acid-deficient diet induced persistent diestrus. The individual estrous cycle patterns of rats that were fed the ThrDef diet are depicted in (A). The triangles indicate each 4-day estrous cycle, beginning with estrus. The gray circles indicate the first day when the estrous cycle delay was observed, and black rectangles indicate the day when continuous diestrus started. (B) shows the average time until the estrous cycle delay was first observed, and the time until continuous diestrus started due to the essential amino acid-deficient diet. The data are presented as the mean±SEM. *significantly different (*P*<0.05) from the ValDef group, and †significantly different (*P*<0.05) from the MetDef and TrpDef group. N = 4–6.

All of the animals in the control group displayed a completely normal 4-day estrous cycle throughout the experiment. The feeding of any of the essential amino acid-deficient diets disturbed estrous cycles, and the animals became persistently diestrus. The typical patterns of the estrous cycles of the ThrDef animals are depicted in Figure 3A. Two normal four-day cycles were observed after the dietary changes in all of the rats. Subsequently, one out of six rats showed persistent diestrus, and the other five rats showed irregular cycles followed by persistent diestrus. Figure 3B illustrates the average time required for the estrous cycle delay to occur following the ingestion of each diet and the duration of the continuous diestrus thereafter. The particular missing amino acid in the deficient diet significantly affected the average time that was required to trigger estrous cycle deterioration. The ValDef diet had a greater effect than the other diets, delaying the estrous cycle by 5.7±0.4 days and triggering continuous diestrus for 5.8±1.1 days after changing the diet. The MetDef and TrpDef diets tended to took more time to influence the estrous cycle. The MetDef and TrpDef diets took 7.0±0.7 days and 7.5±0.7 days to delay the estrous cycle and 8.8±1.2 days and 9.7±0.7 days to trigger continuous diestrus, respectively. The ThrDef (10.7±0.7 days to delay the estrous cycle, 12.0±0.8 days to trigger continuous diestrus) and LysDef (13.3±2.6 days to delay the estrous cycle, 18.7±4.5 days to trigger continuous diestrus) diets caused significantly less estrous cycle deterioration than did the ValDef diet.

Table 4 presents the effects of caloric restriction alone on the estrous cycle in the pair feeding experiment. Although the Pair-fed-100% group was fed the same amount of control diet as the ThrDef group, their average body weight was significantly greater than the ThrDef group (Fig. 2) and their estrous cycles showed consistently perfect four-day cycles throughout the experiment. In the Pair-fed-66% group, two of the six animals displayed one five-day estrous cycle, while the other four animals displayed a regular 4-day cycle during the experimental period. No persistent diestrus was observed in any rats in this group. The average body weight of this group was similar to that of ThrDef rats. In the Pair-fed-33% group, a disturbance of the 4-day cycle was observed 11.7±0.67 days after the dietary change, and the effect subsequently shifted to persistent diestrus, which was a response similar to that of the ThrDef group.

**Table 4:** The estrous cycle in Wistar-Imamichi rats fed each experimental diet.

	The 1st day when	The day when
	the delay of	continuous diestrus
	estrous cycle started	started
Control	-	-
ThrDef	10.7±0.7	12.0±0.8
Pair-fed-100%	-	-
Pair-fed-66%	*	-
Pair-fed-33%	11.7±0.7	12.8±0.2

*Two of 6 rats showed only one extended estrous cycle (5-day) during this period.

The data are present as mean±SEM.

In the re-feeding experiment, the control diet increased the animals' food intake and body weights on the first day after the dietary change in both the ThrDef-4-day and the ThrDef-14-day groups. Estrous cycle recovery, as monitored by vaginal smears, is presented in Table 5. The persistent diestrus caused by the ThrDef diet was reversed by control diet re-feeding in all animals. The duration of ingestion of the ThrDef diet significantly affected the time required for the control diet to restore a normal estrous cycle. The ThrDef-14-day group took 3–4 days longer than the ThrDef-4-day group to recover a normal estrous cycle.

**Table 5:** Control diet consumption restored the normal 4-day estrous cycle in threonine deficient Wistar-Imamichi rats.

	The 1st day when	The day when
	the estrous cycle	the 4-day estrous
	re-started	cycle re-started
ThrDef-4day	3.6±0.2	4.6±1.2
ThrDef-14day	6.3±0.4*	8.6±1.3*

The data are present as mean±SEM.

*Significantly different (P<0.05) between the ThrDef-4day and ThrDef-14day groups.

### Amino acid levels in the plasma and ovaries

**Figure 4:**
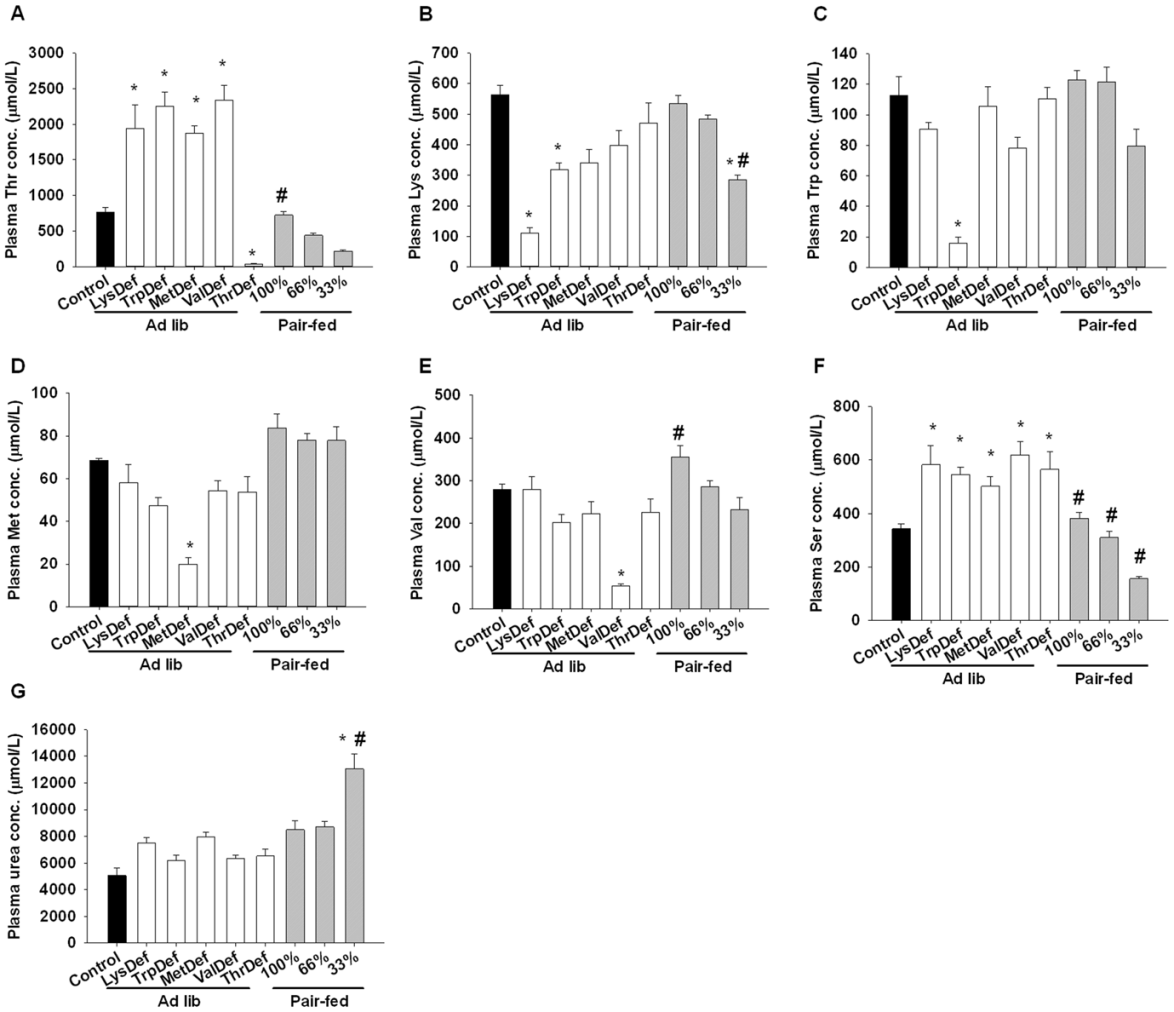
Essential amino acid-deficient diets perturbed plasma aminograms. Plasma threonine (A), lysine (B), tryptophan (C), methionine (D), valine (E), serine (F) and urea (G) concentrations in rats that were fed one of the experimental diets. The data are presented as the mean±SEM. *significantly different (*P*<0.05) from the control group and #significantly different (*P*<0.05) from the ThrDef group. N = 4–6.

The plasma amino acid concentrations after the dietary changes are illustrated in Figure 4. A one-way ANOVA showed significant differences in the plasma levels of Thr (F = 39.6, *P*<0.001), Lys (F = 16.5, *P*<0.01), Trp (F = 19.7, *P*<0.01), Met (F = 15.6, P<0.01), Val (F = 15.3, *P*<0.01), Ser (F = 16.0, *P*<0.01) and urea (F = 16.0, *P*<0.01) under each nutritional condition. Among these amino acid levels, only Ser and Thr levels were significantly elevated in essential amino acid-deficient groups compared to control rats. The plasma Thr levels were significantly higher in the LysDef, TrpDef, MetDef and ValDef groups, whereas the Thr levels in ThrDef rats were lower. The plasma Ser levels were significantly upregulated in all essential amino acid-deficient groups compared to the control rats. The plasma levels of Lys, Trp, Met and Val were decreased after rats were fed the LysDef, TrpDef, MetDef and ValDef diets, respectively. The plasma urea concentration was significantly higher in the Pair-fed-33% group than in control rats.

**Figure 5:**
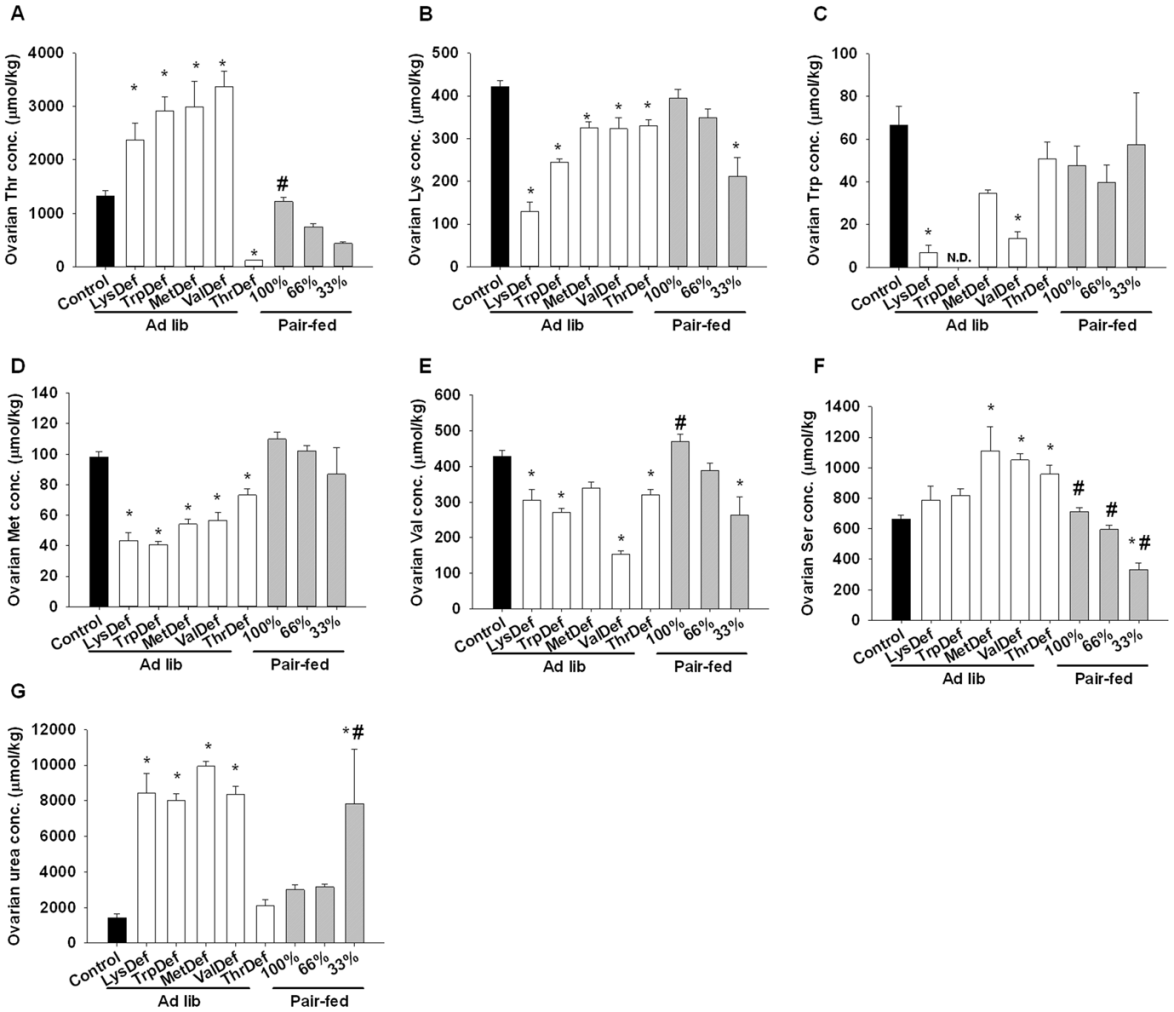
Essential amino acid-deficient diets perturbed ovarian aminograms. Ovarian concentrations of threonine (A), lysine (B), tryptophan (C), methionine (D), valine (E), serine (F) and urea (G) in rats that were fed one of the experimental diets. The data are presented as the mean±SEM. *significantly different (*P*<0.05) from the control group and #significantly different (*P*<0.05) from the ThrDef group. N.D., not detected. N = 4–6.

The amino acid levels in the ovaries are depicted in Figure 5. A one-way ANOVA showed significant differences in the levels of Thr (F = 41.0, *P*<0.01), Lys (F = 25.1, *P*<0.01), Trp (F = 4.5, *P*<0.01), Met (F = 20.2, P<0.01), Val (F = 27.2, *P*<0.01), Ser (F = 19.5, *P*<0.01) and urea (F = 7.9, *P*<0.01), depending on the nutritional conditions. Among these amino acid levels, those of Thr, Ser and urea were significantly elevated in specific essential amino acid-deficient groups compared to control rats. The alterations in ovarian Thr levels were similar to those in the plasma, and significantly higher concentrations were observed with the LysDef, TrpDef, MetDef and ValDef diets, whereas ThrDef rats had significantly lower levels. Ovarian Ser levels were significantly upregulated only in the ValDef and ThrDef groups. The changes in ovarian urea levels were unique. Rats that consumed the LysDef, TrpDef and ValDef diets had markedly elevated ovarian urea levels, but the ThrDef diet did not affect this parameter. The Pair-fed-33% group also had elevated ovarian urea levels. The ovarian levels of Lys, Trp and Val were significantly decreased by feeding the LysDef, TrpDef and ValDef diets, respectively.

## Discussion

This study found that consumption of a Val, Met, Trp, Thr or Lys-deficient diet promptly induced a persistent diestrus state in female Wistar-Imamichi rats. This persistent diestrus or anovulatory state was nutritionally reversible and could be restored by the consumption of a control diet. This disturbance in the cycle was triggered not by decreased caloric intake but by the various single essential amino acid deficiencies because pair-fed counterparts, which were fed identical amounts of an isocaloric control diet, maintained regular estrous cycles. The length of time required to achieve the diestrus state varied among the amino acid-deficient diets: the ValDef diet took the shortest period of time, and the LysDef diet took the longest period of time.

We previously observed that dietary essential amino acid deficiency decreased food intake remarkably in male rats or mice [29], [30], [36], [37]. This reduction in food intake was also observed in this study among female Wistar-Imamichi rats (Fig. 1B) and resulted in an overall loss in body weight (Fig. 1A). Anorexia is a characteristic phenomenon that is induced by many types of dietary essential amino acid deficiencies. A valine deficiency is one of the strongest inducers of anorexia among deficiencies of the 9 essential amino acids, while a lysine deficiency has the mildest effect [36]. In the experiments using male rodents, the food intake of male mice that are fed a ValDef diet declines to 33% of the control level and is associated with a decrease in body weight [37], which is consistent with the data obtained from male rats that are fed a ValDef diet [29]. It was recently reported that a chemosensor in the anterior piriform cortex of the brain, which surveys the balance of dietary amino acids, is involved in the central mechanism that is responsible for the rejection of diets that are imbalanced or deficient in essential amino acids [38], [39]. An amino-acid-deficient or amino acid-imbalanced diet causes a specific increase in uncharged tRNAs of the relevant amino acid, which activates general control nonderepressible 2 in the anterior piriform cortex [38], [39]. Although the mechanism by which the hypothalamus regulates food intake under an essential amino acid deficiency not clear, hypothalamic somatostatin has been shown to be involved in this regulation [37]. The female control rats showed a clear 4-day feed intake cycle that was synchronized with their estrous cycles, i.e., food intake was the lowest on the day of estrous (Fig. 1B). Not only was a significant repression of food intake observed after the onset of all of the essential amino acid-deficient diets, but cyclic changes in food intake were also not detectable (Fig. 1B). Because the magnitude of the anorectic effect of feeding any one of essential amino acid-deficient foods did not differ between male and female rats, male and female rats may have similar central mechanisms of regulation of food intake.

In this study, the reduction in body weight is believed to result from a decline in caloric intake and an enhancement of endogenous protein catabolism, which is induced by the amino acid imbalance [40], [41]. Diets that lack only one of the essential amino acids decrease the plasma level of the deficient amino acid and induce a number of metabolic changes [30], [36], [42], [43]. A decrease in the plasma amino acid level predisposes tissues toward proteolysis to release the deficient amino acid from endogenous proteins, yielding all 20 proteinogenic amino acids [40], [41]. In addition, experiments performed in mice also demonstrated that the decline in food intake and body weight by dietary valine deficiency was restored in a dose-dependent manner by valine supplementation [37]. These findings suggest that the changes in food intake and body weight are solely attributable to the amino acid deficiency. Consistent with the disappearance of the food intake cycle, a body weight fluctuation due to cyclic changes in food intake was not observed after feeding one of essential amino acid-deficient diets (Fig. 1A). The anorexia induced by an essential amino acid-deficient diet is not as detrimental as that resulting from the ratio of preference relative to the control diet. Instead, it serves as a form of protection against protein catabolism in the body. When presented with other foods following the rejection of such a diet, animals began foraging for a diet that is balanced in essential amino acids, and the animals developed a conditioned aversion to cues that were associated with the deficient diet [29], [37].

Reproductive functions are inhibited by severe caloric restriction [10], [11], [12], while moderate caloric restriction can extend the reproductive lifespan [4], [18]. The cessation of estrous cycles in this study (Fig. 3), however, could not be attributable to the anorexia or the loss of body weight that followed. Pair-fed groups that were fed an isocaloric control diet were used. The Pair-fed-100% group, whose caloric intake was adjusted to be equal to that of the ThrDef group, maintained regular 4-day estrous cycles throughout the experiment (Table 4). The Pair-fed-66% group, whose caloric intake was restricted to two-thirds of the intake of the ThrDef group, lost a similar amount of body weight as the ThrDef group. Four out of the six rats continued regular four-day cycles, while the four-day cycles in the other two rats were interrupted once by a five-day cycle (Table 4). The robustness of the cyclicity in the pair-fed groups suggests that the cessation of the estrous cycle was attributable to the deficiency of the essential amino acid itself. These pair feeding experimental results are consistent with the results of previous studies showing that restricting food intake in female rats to 50–60% of ad libitum intake does not significantly alter their estrus cycles [4], [18]. McShane et al. [4] report that the average estrous cycle was greater in length in rats under this magnitude of caloric restriction compared to those that were fed ad libitum during the first 3 months of moderate caloric restriction but did not differ thereafter. Pair-fed-100% and Pair-fed-66% groups in this study consumed approximately 60% and 40% of the ad libitum intake, so the elongation of the estrous cycle to 5 days, which was observed in two rats in Pair-fed-66% group, was consistent with the results of this study. Furthermore, because essential amino acid-deficient groups, whose estrous cycles were completely interrupted, consumed between 44% (ValDef diet) and 68% (LysDef diet) of the control rat intake, the disturbances of the estrus cycle can be considered to be due to the deficiency of the amino acid itself in the diet. However, lengthening and cessation of estrous cycles occurred in the Pair-fed-33% group. This irregularity was most likely due to their highly restricted caloric intake and reduced availability of oxidizable metabolic fuels [11], [12], [16], [17], [18].

To determine the effects of a single essential amino acid deficiency on basic biochemical parameters, we measured hepatic triglyceride levels and plasma glucose, total cholesterol, triglyceride and NEFA levels (Tables 1 and 2). In contrast to the remarkable decreases in body, total fat and liver weights, these biochemical parameters were fairly stable during the consumption of any single essential amino acid-deficient diets, suggesting the involvement of strong homeostatic regulation of glucose and lipid metabolism under these dietary conditions. Only plasma triglyceride levels were generally lowered, and significant decreases in the plasma glucose level were observed in the ThrDef, MetDef and ValDef groups (Table 1).

On the other hand, the plasma levels of amino acids were markedly affected by the feeding of single essential amino acid-deficient diets (Fig. 4). Consistent with the deficiency, Thr, Lys, Trp, Met and Val levels were decreased by the consumption of diets that are deficient in those nutrients. We previously reported that single essential amino acid-deficient diets specifically elevate the plasma serine and threonine concentrations in male rats, reflecting hyper-production of serine and threonine by hepatocytes. The expression of 3-phosphoglycerate dehydrogenase, the rate-limiting enzyme for serine synthesis, is upregulated, while serine dehydratase, which catalyses the conversion of serine and threonine to pyruvate and alpha-ketobutyrate, respectively, is downregulated [30], [36]. These elevations in plasma serine and threonine levels were reproduced in this study using female rats (except for Thr levels in ThrDef rats), whereas rats in the pair feeding groups, whose dietary amino acids were balanced, did not show elevated levels. Therefore, the impacts of caloric restriction and single essential amino acid deficiencies on amino acid metabolism seem to be very different. The extent of weight loss in the ovaries was comparatively minor (Tables 1 and 4), and the free amino acid profile in ovarian tissues generally followed that seen in the plasma (Fig. 5). Several amino acid transport systems were found to be active in oocytes during growth and maturation [44]. Thus, theoretically, an amino acid imbalance in the ovaries could prevent oocytes from maturing, stop ovulation and cease the estrous cycle.

In this study, the plasma levels of IGF-1, leptin and insulin were markedly reduced, while the plasma level of desacyl ghrelin was elevated in rats that were fed an essential amino acid-deficient diet (Table 3). LH secretion is principally stimulated by gonadotropin-releasing hormone (GnRH) in the hypothalamus. GnRH synthesis in GnRH neurons is directly or indirectly controlled by many circulating hormones, including estrogen, leptin, insulin and ghrelin. Leptin knockout (ob/ob) or leptin receptor knockout (db/db) mice display reproductive deficits and infertility. The physiological conditions of low circulating leptin levels during negative energy balance usually induce the suppression of LH secretion and fertility, and leptin administration during negative energy balance restores LH levels and reproductive function [12]. The low level of circulating leptin is considered to be due to reduced body fat content, and it may partly cause the deterioration of estrous cycle under conditions of dietary amino acid deficiency. However, because the body fat content of pair feeding groups is also significantly reduced compared to control rats, it is likely that circulating leptin is decreased in these groups as well. The unique alteration induced by dietary essential amino acid deficiency is the marked reduction in the IGF-1 level. The dietary essential amino acid deficiency suppresses hepatic IGF-1 mRNA expression [33], [34]. The decline in circulating IGF-1 level hinders the correct proliferation of the lumen epithelium in the uterus and the progression of the estrous cycle [27]. Further studies should be performed to uncover the underlying mechanism that induces the cessation of the estrous cycle.

Another important finding of this study is that the estrous cycle perturbations were reversible (Table 5). Although the duration of time that the ThrDef diet had been ingested (4 vs. 14 days) significantly affected the time required to restart a normal 4-day estrous cycle, the diestrus induced by the consumption of the ThrDef diet for two weeks was reversible by the consumption of the control diet for 6.3±0.4 days. The estrous cycle then reappeared in all animals. Rats and mice are thought to be grain eaters by nature, implying that the lysine content of their diet might be extremely limited and that they could have difficulties finding amino acid-balanced diets over the long term [45]. With a shortage of well-balanced amino acid sources, reproduction becomes risky for both the mother and the fetus [46], [47], [48], [49]. Continuous diestrus could be viewed as an adaptation to nutritionally imbalanced conditions, diverting resources away from reproduction and reallocating them to survival until well-balanced amino acid sources are found. Nutritionally reversible cessation of the estrous cycle could be an advantageous adaptation that balances reproduction and survival in rodents.

Gender-specific nutrition is one of the most important topics of research in modern health care. In particular, understanding the nutrients that women require to experience normal menstrual cycles is vital. Stress-induced amenorrhea, which is usually called hypothalamic amenorrhea, affects a consistent percentage of women, regardless of their age [50]. Undernutrition, excess physical training and psychological stress are factors that can potentially trigger hypothalamic amenorrhea, which leads to decreased estrogen levels due to reduced ovarian activity [50], [51]. In addition, women with anorexia nervosa are known to exhibit amenorrhea [6], [7]. Considering the results of this study, negative energy balance and amino acid imbalances could play pivotal roles in amenorrhea.

## Materials and Methods

### Animals and diets

The entire experiment was reviewed and approved by the Animal Committee for animal care at Fukui University (permit number for this study: 22018). Six-week-old female Wistar-Imamichi rats were purchased from the Institute for Animal Reproduction (Ibaragi, Japan). The animals were housed individually in hanging wire cages with free access to water and food. The animal room was maintained at 23±2°C with a 12 h light/dark cycle (lights on 8:00−20:00). The rats were adapted to the cage for one week after their arrival. Food intake and body weight were measured, and vaginal smears were observed between 10:00 and 11:00 every day. The estrous cycle consists of four stages, termed proestrus, estrus, metestrus and diestrus, and it is exactly four days long in Wistar-Imamichi rats. The stage of estrous was determined by cytological evaluations of the vaginal smears [52].

In the pre-experimental period, rats were offered a normal laboratory pellet diet (NMF, 3.49 kcal/g; Oriental Yeast Co., Ltd., Tokyo, Japan) for at least 8 days followed by the control diet for at least 8 days. The control diet was based on the modified AIN93G standard diet but replaced the casein portion with an equivalent free amino acid mixture that provided the entire dietary nitrogen source (Table 6). During this period, estrous cycle regularity was confirmed by monitoring vaginal smears. The plasma amino acid profile of rats that were fed the control diet was found to be similar to that of rats that were fed the AIN93G standard diet. Animals that showed four continuous 4-day estrous cycles, i.e., twice under NMF and twice under the control diet, were used for the experiment. Rats whose estrous cycles were irregular during the pre-experimental period were excluded from the experiment.

**Table 6:** Diet composition.

%, w/w	
Amino Acid Mixture	20
L-Cystine	0.3
Corn Starch	62.9
Cellulose	5
Soybean Oil	7
AIN93G Mineral Mix	3.5
AIN93 Vitamin Mix	1
Choline Bitartrate	0.25
t-Butyl Hydroquinone	0.0014
Energy (kcal/g)	3.9

### Experimental design

Threonine-deficient (ThrDef), methionine-deficient (MetDef), valine-deficient (ValDef), lysine-deficient (LysDef) and tryptophan-deficient (TrpDef) diets were made isonitrogenous using glutamine [29], [30], [36] (Table 7). Of all of the essential amino acids, the diets that were deficient in Thr, Met, Val, Lys or Trp were chosen because the plasma Lys concentration is highly conserved, regardless of nutritional conditions, due to its capacity for reservation and slower catabolism [30], [36]. Conversely, Val deficiency is associated with severe observable phenotypes in rats [29], [37]. Thr deficiency is associated with a marked decrease in the plasma Thr level [42], [43]. Restricting either Trp or Met is reported to affect lifespan extension in rodents [35]. On the morning of metestrus during control diet feeding, the rats were randomly divided into five diet groups: control, ThrDef, MetDef, ValDef, LysDef or TrpDef (N = 4-6 in each group) and were kept on these diets until the end of the experiment. On the 15^th^ day of persistent diestrus, the rats were anesthetized with ether. Blood, liver, ovaries and fat pads were collected. The fat pads were obtained from subcutaneous inguinal, perigonadal and dorsal abdominal fat pads around the kidneys and combined (Tables 1 and 2). The tissues were immediately freeze-clamped and stored at –80°C until analysis. Control rats were euthanized at metestrus when their reproductive stage was most similar to that of the essential amino acid-deficient groups, and their blood and tissues were obtained.

**Table 7:** Amino acid composition in each diet.

%, w/w	Control	ThrDef	LysDef	TrpDef	MetDef	ValDef
Alanine	2.55	2.55	2.55	2.55	2.55	2.55
Arginine	3.28	3.28	3.28	3.28	3.28	3.28
Asparagine H_2_O	3.6	3.6	3.6	3.6	3.6	3.6
Aspartate	3.16	3.16	3.16	3.16	3.16	3.16
Cystin	0.5	0.5	0.5	0.5	0.5	0.5
Glutamine	9.16	11.5	16.22	9.94	10.35	12.74
Glutamate	9.16	9.16	9.16	9.16	9.16	9.16
Glycine	1.62	1.62	1.62	1.62	1.62	1.62
Histidine	2.54	2.54	2.54	2.54	2.54	2.54
Isoleucine	4.45	4.45	4.45	4.45	4.45	4.45
Leucine	8.13	8.13	8.13	8.13	8.13	8.13
Lysine • HCl	8.82	8.82	0	8.82	8.82	8.82
Methionine	2.43	2.43	2.43	2.43	0	2.43
Phenylalanine	4.5	4.5	4.5	4.5	4.5	4.5
Proline	9.37	9.37	9.37	9.37	9.37	9.37
Serine	5.06	5.06	5.06	5.06	5.06	5.06
Threonine	3.81	0	3.81	3.81	3.81	3.81
Tryptophan	1.08	1.08	1.08	0	1.08	1.08
Tyrosine	4.85	4.85	4.85	4.85	4.85	4.85
Valine	5.73	5.73	5.73	5.73	5.73	0
Amino Acids-Total	93.82	92.35	92.06	93.51	92.58	91.67
Starch	6.18	7.65	7.94	6.49	7.42	8.33
Total	100	100	100	100	100	100

Three groups of rats underwent a pair feeding experiment. On the morning of metestrus during the control diet feeding, these rats were divided into three diet groups: 100% (Pair-fed-100%), 66% (Pair-fed-66%) or 33% (Pair-fed-33%) pair-fed (N = 4–6 in each group). Pair-fed-100% rats were offered the same amount (g) of control diet as the ThrDef group had spontaneously ingested, Pair-fed-66% rats were fed two thirds as much as the ThrDef group, and Pair-fed-33% rats were fed one third the amount that the ThrDef group had spontaneously ingested. At the end of the experiment, blood and tissues were collected.

In the third re-feeding experiment, two groups of rats were used. On the morning of metestrus during the control diet feeding, all rats were given the ThrDef diet. Within several days, persistent diestrus was observed by vaginal smears. On either the 4^th^ (ThrDef-4day) or 14^th^ (ThrDef-14day) day of persistent diestrus, the rats were re-fed the control diet (N = 6 in each group). This experiment was continued until the recovery of a normal estrous cycle was observed by the criteria of two continuous 4-day estrous cycles. At the end of the experiment, the rats were euthanized by deep anesthesia. No mortalities were observed during any of the studies.

### Amino acid analyses

Plasma samples were mixed with 2 volumes of 5% (w/w) trichloroacetic acid, centrifuged (4°C, 15 min, 10,000 x *g*) to remove the precipitate and were filtered through a Microcon Ultracel YM-10 (Nihon Millipore, Tokyo, Japan). To measure the amino acid levels in the ovaries, the ovaries were rinsed well in saline to minimize contamination by extracellular fluid or plasma. Ovarian samples were homogenized, deproteinized in 10% (w/w) trichloroacetic acid and filtered. The amino acid concentrations in these filtrates were measured by an automatic amino acid analyzer (L-8800A; Hitachi, Tokyo, Japan). Briefly, amino acids that were separated by cation-exchange chromatography were detected spectrophotometrically after a postcolumn reaction with a ninhydrin reagent [30], [36], [53].

### Analyses of biochemical parameters in plasma and liver

The plasma levels of glucose, total cholesterol and triglyceride (Tables 1 and 2) were determined enzymatically using an autoanalyzer (Fuji Dry-Chem 7000, Fujifilm Medical, Tokyo, Japan). The plasma concentrations of nonesterified fatty acids (Tables 1 and 2) were enzymatically determined using a colorimetric method from a commercially available kit (NEFA C test, Wako Pure Chemicals, Osaka, Japan).

The liver samples were homogenized with 5 volumes of (w/w) phosphate-buffered saline. The lipids were extracted from the homogenate with isopropanol and centrifuged (4°C, 10 min, 10,000×*g*) to remove the precipitate. The triglyceride contents of these samples were measured using commercially available enzyme reagents (Triglyceride E-test Wako, Wako Pure Chemicals, Osaka, Japan).

### Analyses of plasma hormone levels

Commercially available enzyme-linked immunosorbent assay (ELISA) kits were used to measure the plasma concentrations of insulin (Morinaga Institute of Biological Science, Yokohama, Japan), leptin (Morinaga Institute of Biological Science, Yokohama, Japan), insulin-like growth factor 1 (IGF-1) (Diagnostic Systems Laboratories, Inc., Texas, United States) and desacyl ghrelin (Mitsubishi Kagaku Iatron, Inc., Tokyo, Japan) according to the manufacturer's instructions (Table 3).

### Statistical analyses

Data are presented as the mean±standard error of the mean and were analyzed by one-way analysis of variance (ANOVA). Changes in the amount of food intake and body weight were analyzed by two-way ANOVA. When the ANOVA indicated a significant effect (*P*<0.05), a post-hoc Tukey's test was conducted to determine individual differences.

## Supporting Information

Figure S1 **Food consumption normalized to body weight.** Daily spontaneous food intake of rats that were fed each essential amino acid-deficient diet is normalized to body weight as grams per day per kg of body weight. The data are presented as the mean ± SEM. The significant differences (*P*<0.05) are shown as “a”. Control vs. LysDef, ThrDef, TrpDef, MetDef and ValDef. N = 4–6.
(TIF)
