# Supplementary figures and images for: Dietary Deficiency of Essential Amino Acids Rapidly Induces Cessation of the Rat Estrous Cycle
Source: PLoS One. 2011 Nov 23;6(11):e28136. doi: 10.1371/journal.pone.0028136 (PMC3223240; doi:10.1371/journal.pone.0028136)

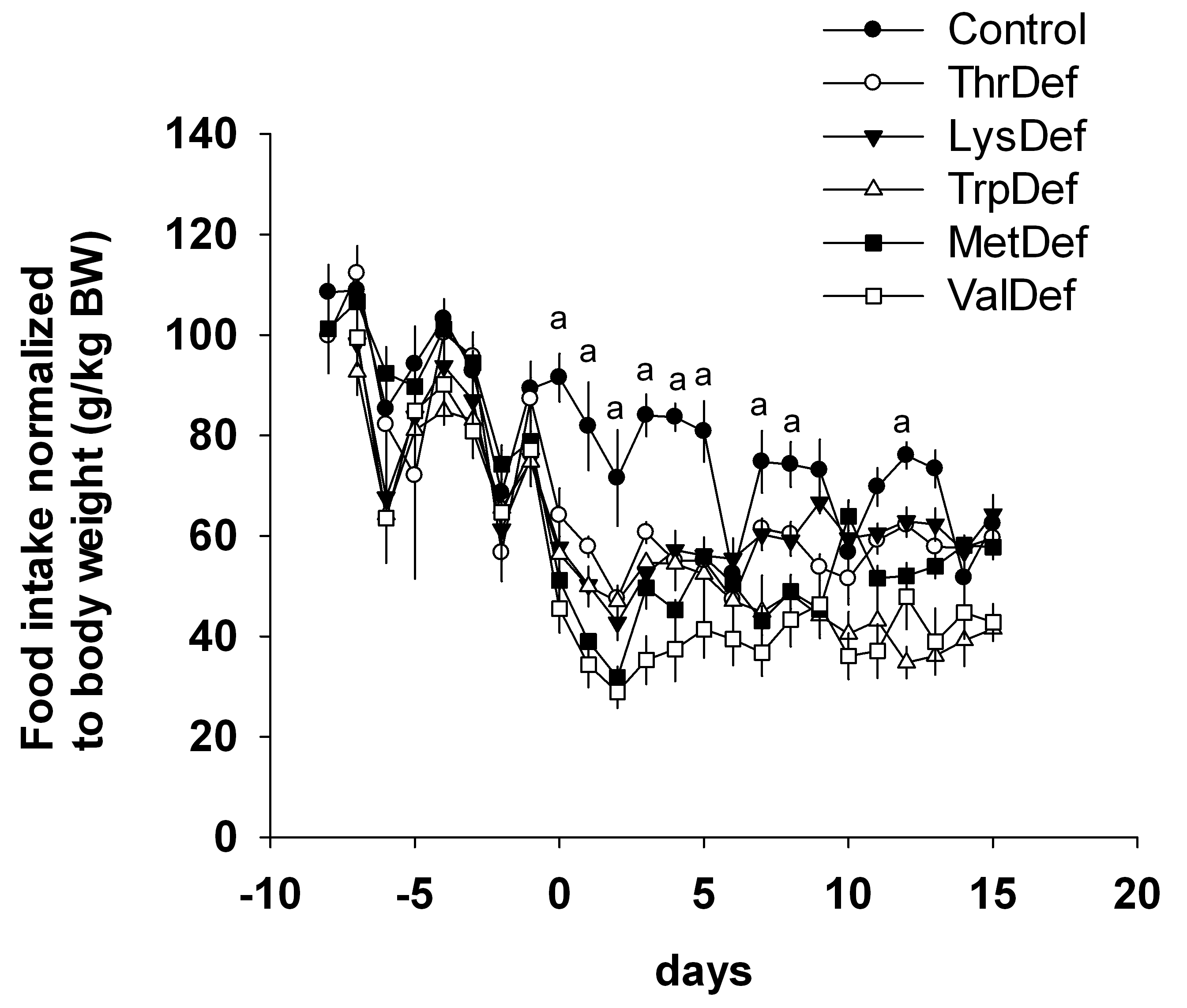

Supplement: Figure S1 — Food consumption normalized to body weight. Daily spontaneous food intake of rats that were fed each essential amino acid-deficient diet is normalized to body weight as grams per day per kg of body weight. The data are presented as the mean ± SEM. The significant differences (P<0.05) are shown as “a”. Control vs. LysDef, ThrDef, TrpDef, MetDef and ValDef. N = 4–6. (TIF) [file pone.0028136.s001.tif]
